# Introduction: the secret lives of microbial mobile genetic elements

**DOI:** 10.1098/rstb.2020.0460

**Published:** 2022-01-17

**Authors:** James P. J. Hall, Ellie Harrison, David A. Baltrus

**Affiliations:** ^1^ Department of Evolution, Ecology and Behaviour, Institute of Infection, Veterinary and Ecological Sciences, University of Liverpool, Liverpool L69 7ZB, UK; ^2^ Department of Animal Plant Sciences, University of Sheffield, Western Bank, Sheffield S10 1EA, UK; ^3^ School of Plant Sciences, University of Arizona, Tucson, AZ 85721‐0036, USA

## 1. Introduction

‘So, Nat'ralists observe, a Flea
Hath smaller Fleas that on him prey,
And these have smaller yet to bite 'em,
And so proceed *ad infinitum*’
∼ Jonathan Swift (1733; see http://www.online-literature.com/swift/3515/)

Early microscopists, training their lenses on samples, observed individual microbes, separated from one another by membranes and cell walls and dividing by binary fission. These observations developed into an understanding of microbial diversity and ecology that, by and large, placed cellular organisms at the centre. How would our perspective of microbiology be different, had those seventeenth century glass grinders invented the genome sequencer instead? A quarter century of using genomics to examine microbial genomes has illuminated a world rife with mobile genetic elements (MGEs): entities that have evolved to persist and replicate through adaptations that move DNA. The roll call of MGEs is long, diverse and growing [1]. Some MGEs, like transposons and insertion sequences, move DNA between locations within a cell. Others, like conjugative elements and bacteriophages, move DNA between cells. Many MGEs are mosaic or modular in their structure, enabling coalitions of different functions and defying straightforward classification [2]. Regardless, it is clear that MGEs can be both powerful and elusive. Unlike the microbes that carry them, it is difficult to meaningfully visualize an MGE, since in most cases, MGEs are essentially strands of DNA, nested within genomes. But as the principal cause of horizontal gene transfer (HGT)—a major mode of microbial evolution in which individual microbes get genetic information from sources other than their parents [3]—MGEs have a huge impact on adaptation and genome structure [4]. Though we have known about MGEs for a long time [5], it is only in recent years, with the explosion of relatively inexpensive sequencing, that we have begun to understand their unique contribution to microbial evolution.

The effective invisibility of MGEs has meant that they have often been conceptualized as traits or properties of their microbial hosts. Many MGEs indeed replicate along with their host cells, and can provide benefits that improve host fitness. But MGEs also have an alternative route to success, because the DNA moved by MGEs often encodes the MGE itself, providing an alternative opportunity for MGE replication that is not necessarily shared with other genes in a cell. As a consequence, these autonomous MGEs experience selection occurring at multiple levels [6]. The resulting pressures may not align with those acting on other regions of the genome, and can instead favour adaptations that can generate intragenomic conflicts [7]. For example, traits that promote selfish MGE transmission to other cells can impose a burden on the current host [8,9]. The evolutionary prospects of an autonomous MGE are thus not confined by the borders of the cell, and instead incorporate transmission across hosts and genetic backgrounds.

One defining feature of living things is their ability to evolve adaptations to enhance reproductive success and fitness. While not alive in any conventional sense, MGEs, with their own interests and adaptive trajectories transcending those of their hosts, might well be considered to have ‘lives’, hidden within the genomic habitat of their host cells. This perspective—viewing MGEs as evolving, self-interested, semi-autonomous actors in their own right—challenges the implicit coherence of the microbial individual, instead revealing genomes to be a contested space of competition and collaboration [10]. What are the implications for understanding MGEs, horizontal gene transfer and microbial evolution?

## 2. In the driving seat: MGEs as vehicles for HGT

MGEs are powerful drivers of HGT, and can have far-reaching effects that extend across microbial communities and beyond, to affect animals, plants, human health and disease, industry and the wider environment. Many ecologically-, clinically- and economically important traits are harboured and transmitted by MGEs, ranging from virulence, to resistance, to bioremediation, to metabolism [11–15]. Indeed, the ability to confer beneficial traits on their hosts is the most conspicuous feature of many MGEs. The effects can be clearly seen in the emerging crisis of antimicrobial resistance, which, to a large extent, is driven by MGEs mobilizing antibiotic resistance genes (ARGs) across species and genera [16]. To understand the propensity for ARG transfer, Wang *et al*. [17] studied the distribution of ARGs across plasmids and chromosomes in three groups of Enterobacteriaceae: *Escherichia*, *Klebsiella* and *Salmonella*. Certain types of resistance gene were more likely to be transmitted by plasmids than others. In particular, genes that caused antibiotic inactivation, or replacement or protection of the antibiotic's target, tended to be more common on plasmids than chromosomes, while resistance genes associated with efflux pumps tended to be more often chromosomally encoded. These patterns reflect broader trends in the susceptibility of genes to undergo HGT, with transfer of genes encoding proteins with multiple interaction partners (such as efflux pumps) proving less successfully transmitted than genes with fewer interactions (such as antibiotic inactivation) [18,19]. The recent global spread of ARGs is perhaps a case study, representative of broader patterns of a general, ubiquitous, ancient process of MGE-mediated gene exchange.

While MGEs *can* spread useful genes, viewing the crucial benefits provided to microbial hosts from the perspective of the MGE itself reveals details and discrepancies that are fundamental to understanding how MGEs cause microbes to adapt. The nitrogen-fixing symbiosis genes of Rhizobia provide an instructive example. In their review on the subject, Wardell *et al*. [20] explain that the complex, multi-component and exquisitely coevolved symbiosis trait is encoded not as part of the core rhizobial genome but is instead located on mobile plasmids or integrative conjugative elements—imposing a fitness cost and impeding co-adaptation with the rest of the genome. Yet this phenomenon makes sense when considering the interests of the MGE and the symbiosis genes it carries: symbiosis mobility enables MGEs to take advantage of patchy selection in the heterogeneous soil environment, to sample different genomic environments, and facilitate competition between different symbiosis elements within the intracellular community.

Taking an MGE's-eye-view to HGT also helps to explain the wide ranging changes to bacterial phenotypes resulting from plasmid acquisition. The accessory genes carried by plasmids can confer distinct, adaptive traits, but often the effects of plasmids extend much further, to significantly influence the expression of diverse resident genes. Billane *et al*. [21] argue that, rather than being maladaptive side-effects, these changes could be considered a manipulation of chromosomal genes to ultimately serve plasmid inclusive fitness. Drawing on diverse examples, including plasmids that rewire core metabolism, promote bacterial virulence, shut down interference competition mechanisms, or trigger biofilm production or motility, they show how plasmid-induced changes in bacterial phenotypes can ultimately favour plasmids, either through vertical or horizontal transmission.

Overall then, though MGEs do act as vehicles of HGT for adaptive traits, they are self-driving autonomous vehicles. The evolutionary destination to which they are being pushed by selection may not be the same as that of their host cells [22]. Instead, MGEs are better conceptualized as fickle symbionts, existing on a continuum, with interactions that range from mutualistic to antagonistic depending on the genetic and environmental contexts [23].

## 3. Contested spaces: conflicts between MGEs and resident genes

MGEs have an intrinsic cost. Under some circumstances, these costs can be outweighed by the benefits of carrying the element, for example, when plasmids harbour accessory genes that are under positive selection [24]. But in most cases, such cargo remains beneficial if it happens to recombine onto the chromosome, leaving a redundant and burdensome MGE [25]. Understanding the source of MGE fitness costs is a major theme in current research. It has long been supposed that the metabolic costs associated with plasmid gene expression, and in particular, translation, make the principal contribution to observed plasmid fitness costs, because of the bioenergetic costs of amino acid biosynthesis and the fact that proteins tend to be more abundant than their corresponding DNA and transcripts [26]. Rodríguez-Beltrán and colleagues [27] addressed the role of translation directly, by examining how plasmid fitness costs are affected in cells with translation defects. If translation were the dominant reason for plasmid costs, then cells with either reduced ribosome elongation rates or fewer ribosomes should suffer exacerbated fitness costs when they are inhabited by a plasmid. But this was not the case, and in fact, impeding translation appeared in some cases to *reduce* plasmid costs. These results suggest that generic mechanisms are less important for plasmid fitness costs than expected, and indicate instead that specific gene conflicts may be the predominant cause, a result that will probably generalize to other types of MGE.

The role of specific genes in mediating the negative effects of plasmids is also supported by a study by Smith and colleagues [28]. Here, the authors examine a curious phenomenon that occurs when *Pseudomonas* species acquire the huge (976 kb) *P. syringae* plasmid pMPPla107: plasmid-carrying cells become sensitive to an unknown factor secreted by competitors during laboratory growth conditions [29]. The evolutionary reason for this sensitivity is unknown, but it can be abolished by evolution occurring in the laboratory, through a single nonsynonymous substitution in the hypothetical plasmid protein SkaA. It is not clear whether or how the wild-type version of *skaA* benefits the plasmid, but the gene appears to be essential for plasmid replication. Regardless, the fact that single basepair mutations can have such dramatic effects on costly plasmid-conferred phenotypes speaks to the importance of specific gene functions, and gene interactions within the context of a host cell [18,30]. With large MGEs carrying huge numbers of genes of unknown function and activity, there is great potential for conflicts to arise, and likewise for new adaptive phenotypes to emerge.

Compensatory evolution can occur to resolve MGE-associated fitness costs [8]. If such mutations occur to chromosomal genes, then microbes that have compensated otherwise costly transmissible MGEs potentially have a double advantage: First, as the costs of MGE carriage are reduced, compensated cells benefit from enhanced absolute growth rates. And second, transmission of the costly MGE into uncompensated neighbours can increase growth of the donor strain relative to those competitors. In this case, the MGE would effectively act as an agent of ‘spiteful’ interference competition [31], a phenomenon explored by Domingues *et al*. [32]. Using a theoretical and individual-based modelling framework, they show that compensated plasmid donor cells may indeed use plasmids as weapons during competition within structured communities. In this context, it is interesting to consider that MGEs have provided the raw material for the evolution of other mechanisms of interference competition, including contact-dependent (type IV secretion) and contact-independent (tailocin) toxins [33,34].

The burden imposed by MGEs drives the emergence of genome defences, which target horizontally-acquired DNA to prevent its establishment [35]. CRISPR-Cas adaptive immunity is a common defence system in bacteria, and is a particularly rich source of information because CRISPR arrays present specific sequences matching their targets, often identifying the antagonising MGE [36]. Pursey *et al*. [37] interrogated a large genomic dataset to investigate whether, in repelling incoming MGEs, bacteria encoding CRISPR were also cutting themselves off from the flow of genetic information via HGT. Overall, they identified a negative association between ARGs and CRISPR-Cas, consistent with a model in which genome defences target vectors of ARGs, but in environments where antimicrobial resistance is beneficial, cells compromise their defences and lose CRISPR-Cas to gain the survival benefits of resistance. It could be that recent strong antibiotic selection has thus rendered many genomes prone to MGEs—an ‘immunocompromised state’ of which MGEs have taken advantage, undergoing rampant transmission, shaping and reshaping microbial genomes in their wake [38]. By opening up genomes to MGEs, anthropogenic antibiotic use may have radical effects on microbial genome evolution beyond simply promoting antimicrobial resistance.

The power of MGEs to drive large-scale genome evolution is explored in the context of transposable elements (TEs) by van Dijk *et al*. [39] in a series of individual-based models that investigate the relationship between TEs and genome streamlining. TEs can wreck their host cells if they insert into an essential gene, and thus highly streamlined genomes are exceptionally susceptible to damaging TE activity. This susceptibility is bad for individual cells, but tends to purge populations of TEs, because TEs also lose out if their host microbe is killed. On the other hand, genomes with redundancy, for example with gene duplications, provide a safer habitat for TEs, because transposition is less likely to disrupt essential genome functions. The predicted consequences are a rock–paper–scissors dynamic that can explain long-term associations between TEs and their hosts, as well as broader patterns in genome evolution and compactness.

The conflicts between MGEs and resident microbial genes thus extend beyond the generic metabolic burdens of MGE gene expression, and instead emerge from particular functions and evolutionary trade-offs. Characterizing these conflicts, and how they are resolved, will help explain patterns of gene acquisition and loss, and identify key species responsible for harbouring and disseminating MGEs in communities.

## 4. MGEs in an MGE world

An MGE is seldom alone. Indeed, MGEs live in a world inhabited by other MGEs, and the genome might well be envisaged as an ecosystem in itself, with many co-existing and interacting agents. Many plasmids, for example, inhabit cells with at least one other plasmid [40], and there are diverse mechanisms by which plasmids can interact to affect conjugation, co-infection and fitness costs [41]. The implication of such plasmid ‘co-infection’ is formally investigated in a framework presented by Igler *et al*. [42]. A salient feature of their models is the emergence of frequency-dependent selection, which varies in nature from positive to negative depending on epistasis between the plasmids. Where plasmids reduce one anothers' costs, the co-infected state is favoured, causing rare plasmids to spread, whereas if plasmids exacerbate one anothers’ costs then rare plasmids are suppressed by dominant ones. The fact that the presence of other plasmids in a community can dramatically affect plasmid fate underlines the importance of considering the effects of a plasmid in the context of other MGEs.

Conflicts and collaborations between MGEs also extend across MGE types. In a series of experiments extended and explored with deterministic modelling, Igler *et al*. [43] investigate how conjugative plasmids and integrative prophages affect each other's transmission. Though neither the plasmid nor the phage used in their experiments (lambda and RP4) contained systems known to directly interfere with transfer, they found that prophages limited conjugative plasmid spread directly by killing recipients, and suggest that prophages may also inhibit plasmid entry. By contrast, in environments with high rates of phage infection, plasmids can benefit from the superinfection immunity provided by a prophage, and are likely to evolve higher conjugation rates in response. In diverse ecosystems, phage infection drives microbial ecology [15], and this context is likely to shape the activity and behaviour of many MGEs, both directly and indirectly.

Indeed, some MGEs have adapted to parasitize phage transmission processes for their own advantage. Satellites are unable to package and transmit themselves, and instead hijack the machinery of phages [44]. P4-like satellites parasitize P2-like phages in Enterobacterales, and de Sousa *et al*. [45] perform a comprehensive overview of the abundance and genetic diversity of these distinct, broadly distributed, and ancient family of MGEs, related neither to phages nor to plasmids. They identify P4-like elements in almost a third of Enterobacterales genomes, and show phylogenetically that these elements have proliferated by HGT across species. Hyperparasitism—being parasitic on a parasite—is a major emerging theme in MGE biology, with *Staphylococcus* pathogenicity islands (SaPIs), phage-like elements (PLE) and phage-inducible chromsomal islands (PICI) also manifesting this strategy [46]. In some cases, MGE hyperparasites offer powerful evolutionary opportunities to their microbial hosts by providing some defence against a heavy burden of phage infection [47]. Viewing this relationship from the perspective of the hyperparasite reveals conflicting evolutionary pressures as these semi-autonomous, but heavily dependent, elements co-evolve with their host bacteriophage and their broader genomic milieu.

Pervasive MGE–MGE competition can also cast MGE accessory traits in a new light. Virus–host mutualism, in which viruses confer beneficial traits on their infected hosts, is widespread [48]. One common mechanism, found across bacteria, is where phages encode toxins that facilitate inter-strain interference competition [49]. *Sulfolobus islandicus*, a hyperthermophilic Archaeal denizen of volcanic hot springs, can be chronically infected with the virus *Sulfolobus* spindle-shaped virus 9 (SSV9). Chronic infection imposes only a small fitness cost, because SSV9 reproduces by budding from host cells, without lysis. In fact, in communities, SSV9 provides a competitive advantage over uninfected strains [50]. DeWerff *et al*. [51] demonstrate that this advantage is due to a virus-encoded specific toxin protein that kills competitors. From the perspective of the virus, killing potential hosts seems counterintuitive, but experiments combined with comparative genomics revealed that related spindle-shaped viruses carry different toxin variants, and it was the carriage of a heterologous virus that rendered strains vulnerable to a toxin. This complex network of cross-targeting makes sense in the context of virus–virus competition, with the host cells caught in the cross-fire.

Together, the understanding that MGEs are adapted for interactions with other MGEs, just as much as (if not more than) for dealing with core chromosomal genes, challenges the conception of microbial individuals as the principal agents adapting in microbiomes. Instead, these findings provoke a more pluralistic model, in which chromosomes and MGEs are nodes in an interacting, coevolving network.

## 5. The diversity of the MGE menagerie

By providing a window into the world of MGEs, genome sequencing has cast light on hidden diversity that would otherwise have remained invisible [52]. Santamaría *et al*. [53] focus their attention on the phages of *Rhizobium* species, which remained poorly understood, despite the crucial ecological and economic importance of this nitrogen-fixing genus. Comparative genomics revealed dozens of viral clusters and the widespread presence of prophage in *Rhizobium* across species and geographic locations, indicating that some phages have wide distribution and host range while others remained more tightly associated. Interestingly, only a small proportion of *Rhizobium* prophages were intact, suggesting that within genomes, prophage are subjected to an ongoing process of degradation. Selection at the level of the microbial host often disfavours MGE independence, driving mutations that prevent MGE transmission (such as the loss of genes for packaging) but retaining genes for the benefit of the host [33,54]. As a consequence, such ‘domesticated’ prophage can be shorter than known-to-be active phage. Pattenden and colleagues [55] developed a model that combines genome sequencing data with measurements of growth characteristics, to investigate how the life history traits of host bacteria influence the length and gene content of putative prophages. While fast-growing hosts displayed a clear bimodal distribution in prophage lengths, slower-growing hosts, and those unlikely to be pathogenic, were relatively depleted in the longer, intact prophage and exhibited a lower rate of prophage induction. These properties are likely to reflect the relative stability of those slower-growing strains, which are less likely to experience boom-and-bust growth cycles and the associated stresses. Such patterns illustrate the dynamic evolution of MGEs in the context of organismal genomes, demonstrating how varied selection pressures can dissociate self-reproducing MGEs into constituent modules.

Advances in long-read sequencing have also enabled larger, repetitive MGEs to be completely assembled and studied, and recent years have seen an increase in the identification of super-sized MGEs, including megaplasmids, which in some cases represent megabases of DNA and harbour hundreds of genes [56,57]. With plasmid size spanning three orders of magnitude, what factors drive megaplasmids to become so large, and what are the consequences for microbial genome evolution? In their review of the field, Hall *et al*. [58] suggest that while there is no meaningful size threshold for assigning megaplasmid status, distinct selective pressures can favour and stabilize larger plasmids and their magnified capacity for HGT.

Comparative genomics is likewise uncovering the opportunism and mosaicism that have emerged as a defining feature of MGE evolution. The plasmids of the agrobacteria–rhizobia complex provide a graphic example. Analysing over 4000 plasmid sequences from this agriculturally-, biotechnologically- and ecologically-important group, Weisberg *et al*. [59] focus on the evolution of the characteristic oncogenic plasmids that are associated with plant disease, finding pervasive hallmarks of recombination and reshuffling that have generated plasmids with new combinations of genes, able to confer pathogenicity to new lineages. In particular, the underexplored accessory plasmids of the complex, which are thought to mainly confer catabolic traits, appear to have contributed key regions and genes, accelerating diversification and extending the functions of virulence plasmids.

The diversity of MGEs uncovered to date is likely to represent just the tip of the iceberg, as technological developments, including long read sequencing, contact sequencing and metagenome assemblies continue to reveal and define the biology of new elements [52,57,60,61]. There is surely much to be discovered, particularly from environmental microbes and those yet to be cultured.

## 6. New perspectives on microbial identities

Exposing the secret lives of MGEs, through sequencing, experiments and modelling, is beginning to force a change in the perspective that has dominated microbiology since those first observations of individual microbes hundreds of years ago. Viewing MGEs as more than simply extensions of their host microbes, and instead as adapting, fitness-maximizing agents in their own right, enables us to draw on a wider range of conceptual frameworks for understanding their behaviour. In a thought-provoking opinion piece, Ghaly *et al*. [62] provide three complementary viewpoints for understanding and controlling MGEs in the context of antimicrobial resistance. MGEs can be conceptualized as biological individuals, as pollutants, and as invasive species, with each of these presentations inviting distinct interventions. For example, by viewing MGEs as biological individuals, attempts could be made to target MGE replication specifically through use of conjugation inhibitors or CRISPR, reducing the collateral damage to their host cells. As pollutants, MGEs could be targeted by adapting methods of wastewater treatment. And as invasive species, we might use tactics used in larger-organism ecology to make communities more stable against invasion. Though focused on controlling the spread of resistance, strategies drawing on such alternative perspectives are more widely applicable, for example, because influencing and controlling MGE activity could facilitate bioaugmentation of microbiomes through introduction of beneficial traits [11].

The realization, stemming from those original microscopic studies, that we live in a microbial world pervaded by tiny lives, is a powerful one, and continues to shape our understanding of health, disease and ecosystem function. We believe that understanding MGEs on their own terms, and uncovering *their* ‘secret lives’ in the process, will ultimately spur new ways of studying, influencing, and comprehending microbiomes, and to advance this perspective, we are proud to present this Theme Issue.

## Data accessibility

This article has no additional data.
